# Phytoremediation Potential and Physiological Mechanisms Underlying Metallic Extraction of *Suaeda glauca*, *Artemisia desertorum*, and *Atriplex canescens*

**DOI:** 10.3390/ijerph192316035

**Published:** 2022-11-30

**Authors:** Changming Li, Tianpeng Gao, Xueying Wang, Shipeng Qu, Yingli Yang, Mingbo Zuo, Juan Wang, Haoming Wang, Guixiang Zhou, Yubing Liu

**Affiliations:** 1 College of Chemistry and Chemical Engineering, Qinghai Normal University, Xining 810008, China; 2 Research Center of Pollution Control and Ecological Restoration Engineering in Mining Area of Gansu Province, Lanzhou 730001, China; 3 College of Biological and Environmental Engineering, Xi’an University, Xi’an 710065, China; 4 Xi’an Institute of Environment Sanitation Sciences, Xi’an 710065, China; 5 Gansu Jinchang Environmental Monitoring Center, Jinchang 737100, China; 6 College of Geography and Environmental Science, Northwest Normal University, Lanzhou 730070, China; 7 College of Life Science, Northwest Normal University, Lanzhou 730070, China; 8 College of Biological and Pharmaceutical Engineering, Lanzhou Jiaotong University, Lanzhou 730070, China; 9 Nanjing Institute of Soil Science, Chinese Academy of Sciences, Nanjing 210018, China; 10 State Key Laboratory of Grassland Agro-Ecosystems, Lanzhou University, Lanzhou 730020, China

**Keywords:** phytoremediation, desert, antioxidation, photosynthesis, hydraulics, heavy metals

## Abstract

Mining activities have led to serious environmental (soil erosion, degradation of vegetation, and groundwater contamination) and human health (musculoskeletal problems, diarrheal conditions, and chronic diseases) issues at desert mining areas in northwest China. Native plant species grown naturally in desert regions show a unique tolerance to arid and semiarid conditions and are potential candidates for soil phytoremediation. Here, an ex situ experiment involving pot planting of seedlings of three native plant species (*Suaeda glauca*, *Artemisia desertorum*, and *Atriplex canescens*) was designed to explore their phytoremediation potential and the underlying physiological mechanism. For Zn and Cu, the three plants were all with a biological accumulation coefficient (BAC) greater than 1. For Cd, Ni, and Pb, *Atriplex canescens* had the highest bioaccumulation concentrations (521.52, 862.23, and 1734.59 mg/kg), with BAC values (1.06, 1.30, 1.25) greater than 1, which indicates that *Atriplex canescens* could be a broad-spectrum metal extraction plant. Physiological analysis (antioxidation, extracellular secretions, photosynthesis, and hydraulics) showed that the three desert plants exploited their unique strategy to protect against the stress of complex metals in soils. Moreover, the second growing period was the main heavy metal accumulation and extraction stage concomitant with highest water use efficiency (iWUE). Taken together, the three desert plants exhibited the potent heavy metal extraction ability and physiological and ecological adaptability to a harsh polluted environment in arid desert areas, providing potential resources for the bioremediation of metal-contaminated soils in an arid and semiarid desert environment.

## 1. Introduction

Mining is the main backbone of social economy and the fundamental drives to the industrial development, while mine excavation also causes crucial environmental contamination in the region [1]. Previous studies found that mining activities, such as ore extraction, refining, and transporting of minerals, could release potentially toxic metallic elements (PTEs) to the surrounding agriculture ecosystem [2]. In China, mining areas have corroded almost 40,000 km^2^ of ground, and the contemporaneously discarded mining area is rising by 330 km^2^ per year [3]. PTEs can be transferred and biomagnified through the trophic food chain, giving an ecotoxicological impact to the local area [4]. Hence, the exploration of environmentally and socially sustainable solutions for soil remediation is a future global challenge.

Currently, the predominant remediation technologies for heavy metal pollution include physical, chemical, biological, and combined remediation technologies [5]. For physical remediation, soil replacement (excavation of originally polluted soils with a clean one) and thermal desorption (microwave and far-infrared desorption) are the mainly emerged technologies [5]. Meanwhile, for chemical restoration, electric remediation (electrodialysis, electromigration, or electrophoresis), soil leaching (inorganic solution, chelating agent, surfactant), and stabilization/curing (inorganic binders, organic binders, thermosetting organic polymers, vitreous materials) are the often-used technologies [5]. These methods have the drawbacks of energy-extensive consumption and introduction of secondary contamination while not achieving permanent removal [6]. Bioremediation technologies are a cost-effective, long-lasting, and green-boosted alternative methodologies for heavy-metal-polluted soils. Among bioremediation technologies, although phytoremediation has drawbacks, including leaching and slowing growth of plants, it has the advantages of causing less secondary pollution and being less destructive to soil structure and so on [7]. Zhang et al. examined the growth responses of indoor-planted *Suaeda glauca* and *Arabidopsis thaliana* to Cd, Pb, and Mn and found that *Suaeda glauca* showed better heavy metal tolerant and accumulative abilities compared with *Arabidopsis thaliana* [8]. A similar findings also showed that the plant *Coriaria nepalensis* could form fertility islands, increase the contents of nutrient elements (C, N, and P), and reduce the contents and toxicity of heavy metals (Mn, Cu, Zn, Cd, and Pb) in southwest China [9]. It is effective to screen local target plants for phytoremediation, because these local species can have more reproductive advantages than the introduced species under heavy metal stress [10]. Recently, there has been increasing enthusiasm for screening and planting local heavy-metal-tolerant plants [11,12].

Jinchang, known as the ‘nickel capital’ of China, is the greatest manufacturing base for nickel (Ni) and cobalt (Co) in the arid and semiarid regions in northwestern China [13]. In the past 50 years, the mine industry caused adverse impacts on the environment and even human health in Jinchang City [14,15]. The distribution of Cu and Ni in Jinchang urban and rural soils was mainly originated from atmospheric dust, airborne particles emitted by ore smelting and mining. The concentrations of Cu and Ni were up to 186.15 and 42.44 mg/kg inside the atmospheric dust [15]. Abandoned mine tailings have poor soil structure and lack nutrients, such as high salt concentration, high pH, and low water holding capacity, which is not conducive to plant growth and development. [11]. Therefore, these results suggest that the application of ex situ remediation is a better option to treat a contaminated site in Jinchang.

Excessive heavy metal accumulation in plants affects the structure and function of organelles, causing the disorder of various physiological and biochemical processes [16]. One of the major impacts is the raised reactive oxygen species (ROS), which broadly refers to superoxide radicals (O_2_^−^), hydroxyl radicals (HO^−^), hydrogen peroxide (H_2_O_2_), and singlet oxygens (1O_2_) [17]. Superoxide dismutase (SOD) is the main O_2_ forager, and its enzymatic reaction brings about the H_2_O_2_ and O_2_ formation. Catalase (CAT) decomposes hydrogen peroxide (H_2_O_2_) into hydrogen (H_2_O) and oxygen (O_2_), which mainly occurs in peroxisomes, cytosol, and mitochondria [18]. Peroxidases (PODs) break down peroxide (H_2_O_2_) from the oxidation of cosubstances as phenolic combinations [19]. The activity of antioxidant enzymes in lettuce (*Lactuca sativa*) increases under the stress of Cd and Pb treatment [20]. The MDA content is normally assigned as a reflection of the membrane lipid peroxidation extent, which is a tiny responsive index for the oxidizing injury [21]. 

In the case of excessive intake of heavy metals by plant cells, these detoxifying enzymes can relieve toxicities from stress, and their reactivation activities can be triggered simultaneously. Alternatively, extracellular secretions, such as proline (PRO) and glutathione (GSH), can also ameliorate peroxidation induced by heavy metals [22]. Photosynthesis is a basic plant metabolism, providing plants with in vitro matter, energy, and oxygen, which is highly sensitive to environmental agents, such as heavy metal stresses [23]. The manner in which plant hydraulic properties (Ks) are coordinated within their adaptation strategies in a desert environment among different species has been well documented in desert plants [24,25]. Additionally, adjusting the intrinsic water use efficiency has been widely assumed to eliminate the injury caused by drought [26], but the precise effects on plants’ heavy metal extraction ability and survival remain unknown.

In this study, *Suaeda glauca*, *Artemisia desertorum*, and *Atriplex canescens*, three species of desert plants endemic to arid and semiarid regions, were ex situ planted in pots in the workstation of the Center for Pollution Control and Ecological Restoration of Mining Areas in Gansu province (CPCERMAGP). The substantial residue of mining tailings was evenly mixed into indigenous agricultural soil, which can reduce the concentration of heavy metals and allow for ex situ remediation [27]. Previous works related to a Jinchang mine focused on metal spatial distribution, environmental risk assessment, and the contents of PTEs in a native plant biomass [11,14,15,28,29], but the physiological responses of these desert plants have not been documented, and it is necessary to draw a clear phytoremediation plant profile for desert tailings in arid and semiarid regions of northwest China. In this respect, the targets of this survey were to (1) examine the potential of the aggregation of heavy metals in these three desert plants and (2) assess the physiological responses coupling with their metal accumulation ability. 

## 2. Methodology

### 2.1. Study Zone and Soil Samplings

**Figure 1:**
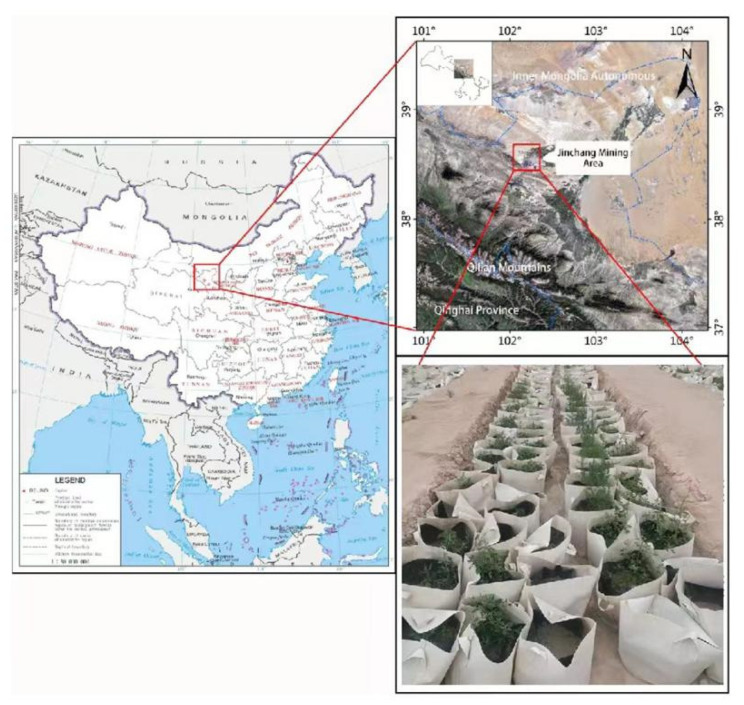
A diagram map of the sampling zone with the planting pots.

The study zone was around the outskirts of Jinchang near a mining area, in the workstation of CPCERMAGP (Figure 1). This area is classified as a normal arid and semiarid region in the northern Qilian Mountains at the southern edge of the Tengger Desert, an area with mean precipitation (104 to 129 mm per year), great evaporation (1900 to 2100 mm), and upper temperatures in the summer (about 22 °C) [29].

The experiment was implemented at an open working station that was 20 km away from the tailings dam in the city of Jinchang. The seeds of desert plants near the mining area were collected in October 2018. In March 2019, the seeds were germinated in the laboratory and then planted in the workstation of CPCERMAGP in an open air and received natural precipitation. Samples of tailings were collected from the deposit of Jinchuan Nonferrous Metals Corporation (JNMC) located in southwestern Jinchang City (Figure 1). The tailings samples were picked up and stored in precleaned airtight plastic bags using a plastic scoop, labelled, and then transferred to the ex situ workstation (CPCERMAGP). Each sample was dealt with separately: one part of them was sent for analysis of the soil’s basic chemical properties and the determination of the content of elementary metal, while another portion was mixed with local original rural soils used for pot planting experiments. The agricultural soil mixed in pot experiments was excavated from the field of Bayi National Farm at a 0–20 cm depth (Figure 1). The original rural soils were obtained from desert soils, and the content was mainly sandy. The rural soils were air-dried and ground, and then passed through a 100 mesh sieve for removing large stones and grass debris.

### 2.2. Pot Planting Experiments in the Field

We collected the seeds of three plants from the irrigated desert regions of the Hexi Corridor, northwest China, to set up the pot experiment. *Suaeda glauca*, *Atriplex canescens*, and *Artemisia desertorum* are typically epidemic plants in desert areas. Without any addition, the original rural soils were put into the control group (CK). The tailings from JNMC were mixed with the original soil at a volume ratio of 1:1 (*v*/*v*) to set the treatment group (T) to simulate ex situ soil restoration. The group settings were identified as follows: CK (original rural soil) and T (tailings with original rural soil). Each pot contained 30 kg total weight of mixed soil, and the basic chemical properties are shown in Table 1. The germinated desert plants were sown in four replications per pot. The changes in physiological indexes were determined for each growing stage: seedling (from germination to growth at 2 months), flowering (from germination to growth at 4 months), and fruiting (from germination to growth at 6 months).

### 2.3. Assertion of Metallic Elements in the Pot Soils and Plant Bodies

Before phytoextraction, soils were tested for extractable metals (cadmium, nickel, copper, zinc, and lead), pH, and cation exchange capacity (Table 1). The extractable metals were collected in 1 M NH_4_NO_3_ and digested following the national standards (State Environmental Protection Administration of China, 1997). The accurate concentration was determined by atomic absorption spectrometry [30]. The background values of the metallic elements in the mine tails are shown in Supplemental Table S1. The pH and cation exchange capacity (CEC) were measured in a 1:5 (*w*/*v*) suspension of soil and water using CyberScan pH 510 digital electrodes (Thermo Scientific, Eutech Instruments Water Quality Analysis Solutions, Shengzhen, China) and an EI 601 digital conductivity meter (Electronics, Beijing, China), respectively [31].

The entire heavy metal concentrations (cadmium, nickel, copper, zinc, and lead) in the whole body of the plants were extracted using an acid digestion mixture (HNO_3_-HClO_4_-HF) (EPA3010A, 1996). The clear solution obtained from the digestion was filtered and reconstituted to the desired volume for analysis using an atomic absorption spectrometer (AAS, M6MK2, Thermo Electron Corporation, Massachusetts USA).

### 2.4. Measurement of Desert Plant Physiological Performance 

Superoxide dismutase (SOD) activity was determined by the method of Beauchamp and Fridovich [32]. Catalase (CAT) activity was determined by the H_2_O_2_ method within a spectrophotometer at 240 nm [30,33]. Peroxidase (POD) activity was measured according to the guaiacol method [30,33]. The content of malondialdehyde (MDA) representing lipid peroxidation was determined using the thiobarbituric acid (TBA) method. The proline (PRO) content was estimated spectrophotometrically from the method described by Bates et al. [34]. Total glutathione (GSH) was measured according to Huang et al. [35].

The net photosynthesis rate (Pn) (μmol CO_2_ m^−2^·s^−1^), transpiration rate (Tr), and stomatal conductance (Cond) were assayed using a portable apparatus (LI-COR 6400XT, Biosciences Inc., Lincoln, NE, USA) at 8:30–11:00 a.m. for each growing stage. The photosynthetic apparatus measured three expanded full leaves (fourth to sixth from the top) per treatment at a light saturation of 1200 μmol m^−2^·s^−1^ [36]. The ambient conditions during the measurements were 400 μmol CO_2_ mol^−1^ air, 24.0 ± 0.2 °C leaf temperature, and 60 ± 5% relative humidity. The measurements in each treatment were performed in triplicate and averaged to represent the data of the treatments. Data for each plant in the treatment were the mean of the three leaf subsamples composing the data of each plant.

The intrinsic water use efficiency (iWUE) was determined as in Formula (1) [37]; the hydraulic conductance (Ks) of plant shoots was measured by the transient and the dynamic modes of a high-pressure flowmeter (HPFM-Gen3; Dynamax Corp., Elkhart, IN, USA) [38].

iWUE = Aarea/Gs (1)

Aarea, the net photosynthesis rate (μmol m^−2^ s^−1^); Gs, stomatal conductance to water vapor (mmol m^−2^ s^−1^). Gs was measured using LI-COR 6400XT, while determining the net photosynthesis rate.

### 2.5. Data Calculation and Statistical Analysis

The biological accumulation coefficient (BAC) for a certain metal equals the metal concentration of the whole body parts (including roots, stems, leaves, and grains) divided by the same metal content in the assigned pot planting soil [39].

Analysis of variance (ANOVA) and Tukey’s multiple range tests (*p* < 0.05) were concerned with the calculation of the statistical significance of the tailings treatment effects on metal concentrations and physiological parameters in SPSS 20 (SPSS Inc., Chicago, IL, USA). Variability in the data was also expressed as the standard deviation of four replicates.

## 3. Results

### 3.1. Heavy Metal Contamination of the Soils

Table 1 shows that the concentrations of five HMs (Cd, Cu, Ni, Pb, and Zn) in the treatment pots are significantly higher than those in the CK soil. The CEC of the soil is lower in the treatment than in the CK, but the difference is not significant. The pH of the soil is almost the same between the CK and the treatment. The concentrations of Cd, Ni, Cu, Zn, and Pb in the treatment pot are 92, 24, 36, 23, and 74 times higher than those in the CK, respectively. The Cd concentration exceeds the risk screening values of the farmland soil pollution control standard (GB15618-2018) [40] by 820 times under treatments. Meanwhile, Ni exceeds the standard by 3.49 times, Cu by 9.38 times, Zn by 4.58 times, and Pb by 8.16 times.

### 3.2. Heavy Metal Concentration in Desert Plants

The heavy metal concentrations of the three plants are presented in Table 2. Almost all five metallic elements in plants increase significantly after the tailings are mixed with native soil compared with the control. The cadmium concentration in the plants varies from 0.05 ± 0.01 to 521.52 ± 0.09 mg/kg, with the maximum value in *Atriplex canescens* under the tailings soil treatment. The nickel concentration in the plants varies from 0.83 ± 0.07 to 862.23 ± 0.04 mg/kg, with the maximum value also in *Atriplex canescens* under the tailings soil treatment. The lead concentration in the plants varies from 0.37 ± 0.03 to 1734.59 ± 0.07 mg/kg, with the maximum value still in *Atriplex canescens* under the tailings soil treatment. Furthermore, there are great variations in metal concentrations among plant species, and the value for copper varies from 0.52 ± 0.09 to 984.90 ± 0.97 mg/kg, while that for zinc varies from 0.59 ± 0.03 to 1454.93 ± 0.91 mg/kg. Among the three plant species, *Atriplex canescens* shows the highest accumulation ability for Ni, Cu, Pb, and Zn. *Suaeda glauca* (Cu, 984.90 ± 0.04 mg/kg; Zn, 1413.74 ± 0.71 mg/kg) and *Artemisia desertorum* (Cu, 966.14 ± 0.60 mg/kg; Zn, 142.47 ± 0.53 mg/kg) also contain higher amounts of Cu and Zn.

Table 3 presents the biological accumulation coefficients (BACs) of the three native desert plants. Of the five metals, plants tend to have stronger Cu and Zn accumulation, and the BAC values of the three plants are all above 1. Meanwhile, for Cd, Ni, and Pb, only the *Atriplex canescens*’s BACs are great than 1. *Suaeda glauca* and *Artemisia desertorum* are below 1 (Table 3).

### 3.3. Physiological Responses of Three Native Desert Plants

**Figure 2:**
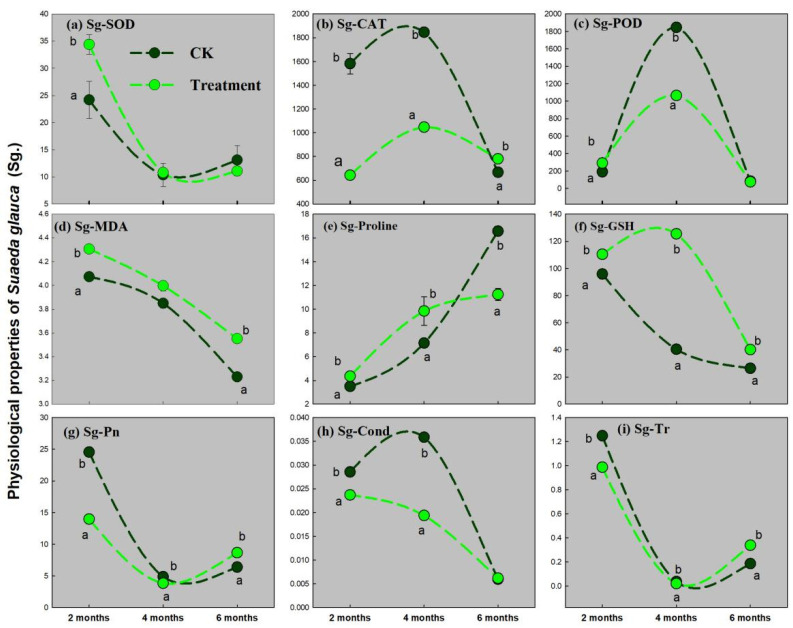
The activity of antioxidase and amount of extracellular excretion in *Suaeda glauca* (Sg.) during three growing periods. (**a**) Superoxide dismutase (SOD), (**b**) catalase (CAT), (**c**) peroxidase (POD), (**d**) malondialdehyde (MDA), (**e**) proline (PRO), (**f**) glutathione (GSH), (**g**) net photosynthesis rate (Pn), (**h**) transpiration rate (Tr), (**i**) stomatal conductance (Cond). Two different lowercase letters (a and b) in the same time point indicate a significant difference (*p* < 0.05) between the treatment and the CK.

**Figure 3:**
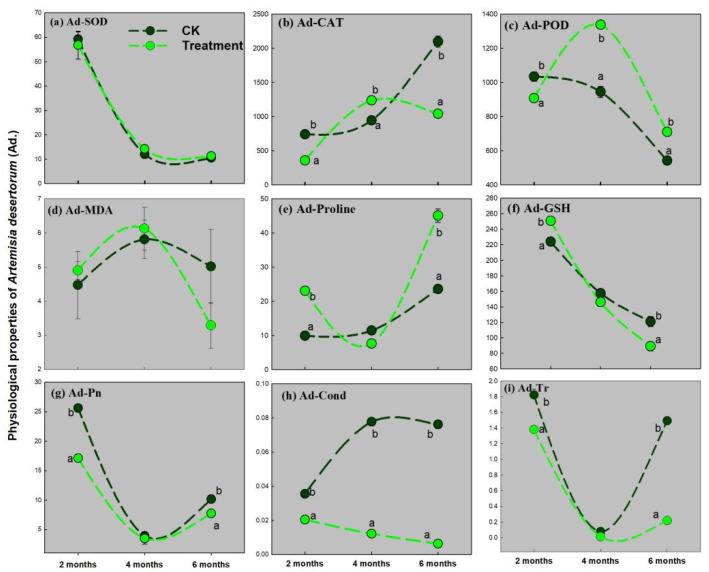
The activities of antioxidase and amounts of extracellular excretion in *Artemisia desertorum* (Ad.) during three growing periods. (**a**) Superoxide dismutase (SOD), (**b**) catalase (CAT), (**c**) peroxidase (POD), (**d**) malondialdehyde (MDA), (**e**) proline (PRO), (**f**) glutathione (GSH), (**g**) net photosynthesis rate (Pn), (**h**) transpiration rate (Tr), (**i**) stomatal conductance (Cond). Two different lowercase letters (a and b) in the same time point indicate a significant difference (*p* < 0.05) between the treatment and the CK.

**Figure 4:**
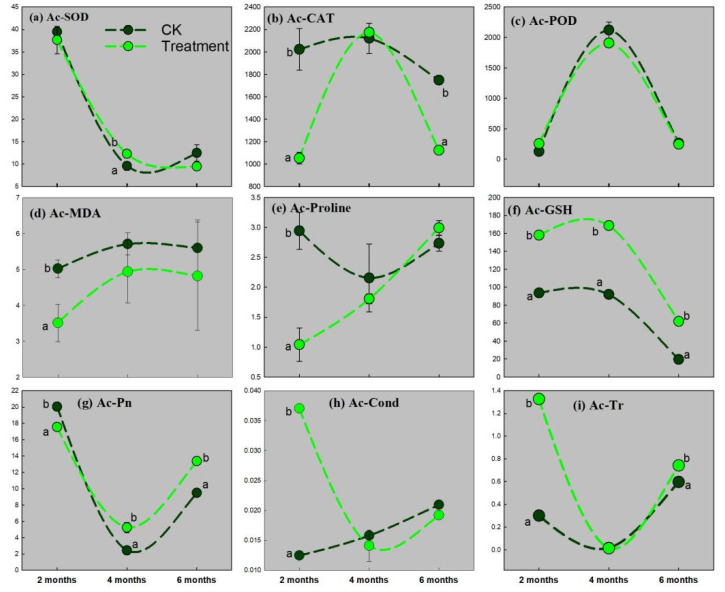
The activity of antioxidase and the amount of extracellular excretion in *Atriplex canescens* (Ac.) during three growing periods: (**a**) SOD, (**b**) CAT, (**c**) POD, (**d**) MDA, (**e**) PRO, (**f**) GSH, (**g**) Pn, (**h**) Tr, (**i**) Cond. Two different lowercase letters (a and b) in the same time point indicate a significant difference (*p* < 0.05) between the treatment and the CK.

Figure 2, Figure 3 and Figure 4 show that the nine physiological indices of *Suaeda glauca*, *Artemisia desertorum*, and *Atriplex canescens* (SOD, CAT, POD, MDA, PRO, GSH, Pn, Cond, and Tr) change during the three growing periods with the pressure of complex heavy metal treatment.

In Figure 2, in the first period, when *Suaeda glauca* grows to 2 months old, the detoxifying enzymes, SOD, extracellular secretion, PRO, and GSH, show to be significantly increasing in reaction to metal stress (Figure 2a,e,f). At the second stage, when *Suaeda glauca* grows to 4 months old, the production of PRO and GSH is more important than the activation of oxidase in answer to the stress of heavy metals in the pot soils (Figure 2e,f). At the last stage, when *Suaeda glauca* grows to 6 months old, only GSH in *Suaeda glauca* is significantly higher in the treatment group versus the control. Pn, Tr, and Cond are all significantly suppressed under heavy metal stress at the first and second growing stages (Figure 2g–i). Meanwhile, Pn and Cond are relived at the last stage. MDA in *Suaeda glauca* is significantly higher in the treatment than that in the CK throughout (Figure 2d).

In Figure 3, extracellular secretion, PRO, and GSH are substantially upregulating in reply to heavy metal stress when *Artemisia desertorum* grows to 2 months old (the first growing stage). Otherwise, oxidase enzyme, CAT, and POD are significantly upregulated in response to heavy metal stress at the second growing stages, when *Artemisia desertorum* grows to 4 months old (Figure 3b,c). At the last growing stages, the PRO and activity of POD are significantly upregulated than that in the CK (Figure 3c,e). MDA is significantly higher under heavy metal stress at the first and second growing stages for *Artemisia desertorum* (Figure 3d). Pn, Tr, and Cond are all substantially suppressed under heavy metal stress throughout the three growing stages (Figure 3g–i).

In Figure 4, there is only GSH upregulated in *Atriplex canescens* under heavy metal stress when it grows to 2 months old. At the second growing stage, the oxidase SOD, CAT, and the extracellular substance GSH in *Atriplex canescens* are upregulated under metal stress, though CAT is not significantly upregulated (Figure 4a,b,f). MDA is always lower in *Atriplex canescens* all throughout the three stages (Figure 4d). Tr and Cond are not suppressed under heavy metal stress at the first *Atriplex canescens* growing stage (Figure 4h,i). Additionally, the suppression of the photosynthetic rate for *Atriplex canescens* is relieved at the second growing stage till the last growing stage (Figure 4g).

### 3.4. Water Use Efficiency and Hydraulic Conductance

**Figure 5:**
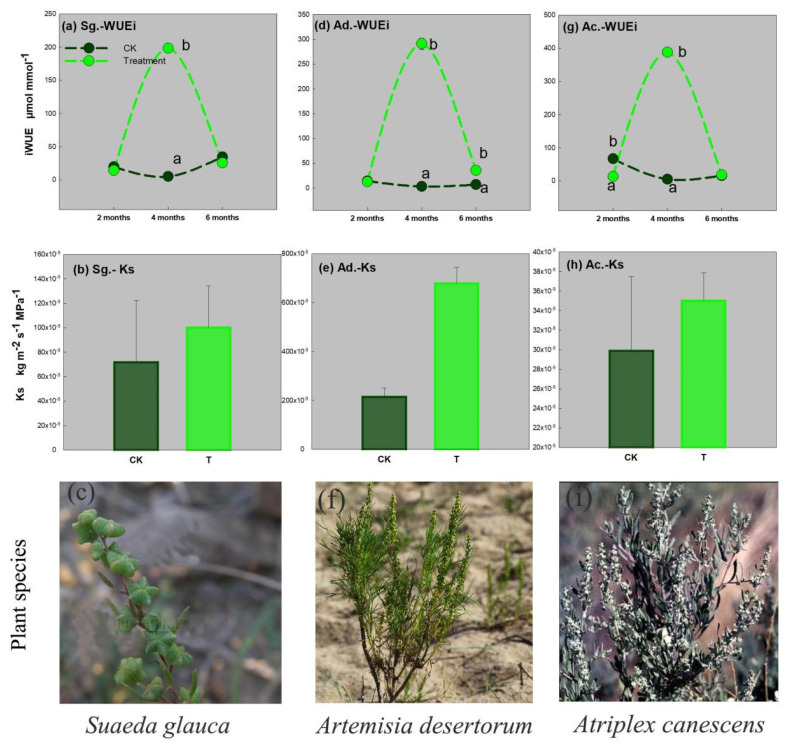
The intrinsic water use efficiency (iWUE) and the hydraulic conductance (Ks) of three native desert plants. (**a**) iWUE of *Suaeda glauca*, (**b**) Ks of *Suaeda glauca*, (**c**) diagram of *Suaeda glauca*, (**d**) iWUE of *Artemisia desertorum*, (**e**) Ks of *Artemisia desertorum*, (**f**) diagram of *Artemisia desertorum*, (**g**) iWUE of *Atriplex canescens*, (**h**) Ks of *Atriplex canescens*, (**i**) diagram of *Atriplex canescens*. Two different lowercase letters (a and b) in the same time point indicate a significant difference (*p* < 0.05) between the treatment and the CK.

In Figure 5, when the three plant grows to the second period (4 months old), the iWUEs of *Suaeda glauca*, *Artemisia desertorum*, and *Atriplex canescens* are significantly upregulated at the pressure of complex heavy metals. The treatment group is 40, 99, and 76 times higher than that in control group, respectively. The iWUE of *Atriplex canescens* is higher than that of *Suaeda glauca* and *Artemisia desertorum* by 1.96 and 1.33 times, respectively. At this stage, under the complex heavy metal stress, the Ks of the three desert plants are upregulated as the result shown in Figure 3b,e,h. However, the effects on the Ks are not shown to be significant.

## 4. Discussion

For the effectiveness remediation of mine-polluted fields, the detection and characterization of native plant species in a mine area are essential to develop phytoextraction technologies for PTE remediation [10,11,12]. Our work selects plants for ex situ phytoremediation of desert mining area soil in western China for the first time. In this study, mine tailings were evenly mixed with local original soil to establish a pot experiment for native desert plant growth. In comparison, as the tillage soil contamination control criterion (GB15618-2018) [40], the focuses on five metals (Cd, Ni, Cu, Zn, and Pb) in the mixed soils all outstripped the risk screening values (Table 1). The concentrations of Pb, Cu, and Zn were at the same degree compared with that of the copper–zinc and lead–zinc mines in Aletai, Xinjiang Uygur Autonomous Region of China [41]. The potentially bioavailable Ni, Cu, and Cd took up about 50–90% of the total metal contents, suggesting that these metals may pose higher ecological threats in soils of the Jinchang urban area [11].

These three plants *Suaeda glauca*, *Artemisia desertorum*, and *Atriplex canescens* were considered to have a higher potential phytoextraction ability to Cu and Zn for their BAC values being all greater than 1. At the same time, Cu and Zn are also trace elements involved in many important biological processes in plants, so the tolerance to Cu and Zn widely exists in various plants [12,42]. Other studies also revealed that *Suaeda glauca* was a good phytoextraction plant at metal-contaminated sites [8,12]. *Atriplex canescens* is expected to be a broad-spectrum heavy metal accumulator species and the highest phytoextraction ability to Cd, Ni, and Pb for its BAC values were more than 1. 

To reveal the heavy metal extraction mechanism at the physiological and hydraulic levels of these native desert plants, we measured their antioxidant enzyme SOD, CAT, and POD activity, the concentration of exocrine gland substances (MDA, PRO, and GSH), the photosynthesis rate, and the hydraulic conductivities during the growing stages. In Figure 2a, the concentration of MDA of *Suaeda glauca* is dramatically upregulated under complex heavy metal stress, suggesting that the plant cell membranes of *Suaeda glauca* encountered oxidative damage caused by complex heavy metals in soils mixed with the tailing soils [8]. ROS accumulation is an ordinary side effect under heavy metal stress, which is the major causes of plant cell membrane lipid peroxidation. SOD is the main O_2_^-^ hunter, and its enzymatic reaction leads to H_2_O_2_ and O_2_ formation [19]. Corresponding to this, exocrine substances, PRO, and GSH were also upregulated in *Suaeda glauca* until the last growing period under heavy metal stress (Figure 2e,f). PRO presents defensive roles for enzymes, membranes, and ROS scavenging in many other plants [43,44,45]. GSH can act as an ancestor to PC synthesis to chelate free metal ions in cytosol with the specific high affinities [46,47]. Pn, Tr, and Cond of *Suaeda glauca* were all essentially smothered at the initial developing phases (Figure 2g–i). This suppression was relieved at the last growing stages, which may be due to the adaptation of *Suaeda glauca* to the stress caused by the complex heavy metals during the whole growing seasons; on the other hand, it may be due to the function of PRO and GSH.

A similar response was observed in *Artemisia desertorum*, in which the concentration of MDA was increased at the first two growing stages, although it did not reach a significant level (Figure 3d). This indicates that under complex heavy metal stress, ROS and lipid peroxidation are triggered in *Artemisia desertorum* during the growing periods. This is different in *Suaeda glauca* and *Artemisia desertorum* upregulated proline and GSH firstly to mitigate this stress under the first growing stages (2 months old) (Figure 3e,f). At the latter stages (4 and 6 months old), antioxidant enzymes, POD, substitute the exocrine substances to act as the ROS scavenging (Figure 3c). POD is broadly dispersed in the plant domain and is a primary enzyme engaged in the disposal of dynamic O_2_·[19]. It assumes a part over diminishing H_2_O_2_ assemblage, MDA elimination, opposing cell peroxidation of membrane lipids, and administering cell layer integument. Pn, Tr, and Cond of *Artemisia desertorum* were fundamentally smothered all around the three developing phases (Figure 3g–3i). This different response to the complex heavy metal stress between *Artemisia desertorum* and *Suaeda glauca* may indicate that the preferential supply of photosynthetic products to the antioxidant system. When the stress of the heavy metals is relieved to some extent by the enzymes, the products of photosynthesis will tap to the production of the exocrine substance, such as PRO and GSH. These two desert plants may coordinate the relationship between the photosynthetic system and the defense system (antioxidant with the exocrine substance) to cope with a complex heavy metal stress environment.

Contrary to *Suaeda glauca* and *Artemisia desertorum*, the concentration of MDA in *Atriplex canescens* was never upregulated under complex heavy metal stress (Figure 4d). Additionally, Pn, Cond, and Tr were not significantly suppressed by the heavy metals all throughout the growing stages (Figure 4g–i). This was different from Cond and Tr in *Suaeda glauca* and *Artemisia desertorum*, which were significantly higher in the complex heavy metal stress than that in the CK at the first growing period. The net photosynthetic rate of *Atriplex canescens* under complex heavy metal stress was significantly greater than that in the CK for the second and last growing stages. That means *Atriplex canescens* may have other ways to solve the problem of heavy metals and to relieve pressure to the photosynthetic system. Sun et al. [48] found that there was a metallic element association protein, AcHMA1, which was upregulated when *Atriplex canescens* was under stress from multiple metals (Cu, Ni, Cd, and Al). This protein could be membrane-restricted and primarily intercede the decontamination of the metal irons. Another reason for *Atriplex canescens* to decouple metal stress was the accumulation of secondary substances, total polyphenols and flavonoids, to the resistance adversity oxidative stress originated from multiple heavy metals (Zn, Pb, and Cd) [49].

Under complex heavy metal suppression, the results of iWUE and Ks showed a similar variation trend, and at the second stage, the iWUEs were sharply increased and the hydraulic conductivity, K value, also had the same upregulating trend in the three native desert plants, *Suaeda glauca*, *Artemisia desertorum*, and *Atriplex canescens* (Figure 5). Less evidence showed that the pathway related to water transport in plants was the main way related to metal ion migration [50]. Going through 4 months of growing at this harsh environment, the plants were gradually adapted to this kind of adversities, and the physiological state had to be most harmonious with the external environment at this stage. In addition, the iWUE was significantly upregulated. These data indicate that the second growing period is the key metal accumulation stage in these desert plants.

## 5. Conclusions

The native species *Suaeda glauca*, *Atriplex canescens*, and *Artemisia desertorum* could be potential candidates for the phytoextraction and phytostabilization of copper and zinc in the arid and semiarid desert mining areas. Three desert plants use their unique mechanism of antimetal toxicity. *Suaeda glauca* tended more to increase the exocrine substances, PRO and GSH, to defend against heavy metal stress. *Artemisia desertorum* tended more to enhance the activity of antioxidizing enzymes, such as SOD and POD, to defend against heavy metal stress. *Atriplex canescens* has its specific ways to protect against complex heavy metal stress through membrane-located proteins and certain secondary metabolites.

The plant photosynthetic system can adjusted to fit its physiological response to complex heavy metal stress. The intrinsic iWUE and Ks were significantly upregulated under complex heavy metal stress at the second stage, in which the main heavy metal stabilization and extraction occurred in these desert plants.

## Figures and Tables

**Table 1:** Metallic elements’ concentrations in original soil (CK) and treatment (T).

	Cadmium	Copper	Nickel	Lead	Zinc	pH	CEC
	mg/kg						Cmol (+)/kg
RV	0.60	100.00	190.00	170.00	300.00	>7.50	
CK	5.35 ± 0.001	26.15 ± 0.01	27.50 ± 0.03	18.70 ± 0.09	58.80 ± 0.04	8.44 ± 0.07	9.32 ± 0.02
T	492.00 ± 0.42	938.00 ± 0.55	663.25 ± 0.60	1387.68 ± 0.57	1372.57 ± 0.75	8.33 ± 0.04	5.80 ± 0.08

Notes: CK is the rural soil sampled without plants in a Jinchang mining area of farmlands; T is the mixture of tailings and agriculture rural soils. CEC is the electrical conductivity. The RV value is the risk screening values for soil contamination of agricultural land in GB15618-2018.

**Table 2:** Heavy Metals enriched in the three desert plant bodies (mg/kg).

	Cadmium (mg/kg)		Nickel (mg/kg)		Copper (mg/kg)		Zinc (mg/kg)		Lead (mg/kg)	
	CK	T	CK	T	CK	T	CK	T	CK	T
*Suaeda glauca*	0.05 ± 0.04 a	123.00 ± 0.03 b	0.83 ± 0.07 a	318.36 ± 0.02 b	0.52 ± 0.08 a	984.90 ± 0.04 b	0.59 ± 0.03 a	1413.75 ± 0.71 b	0.94 ± 0.05 a	693.84 ± 0.07 b
*Artemisia desertorum*	1.39 ± 0.01 a	162.36 ± 0.01 b	12.10 ± 0.03 a	338.26 ± 0.02 b	8.63 ± 0.06 a	966.14 ± 0.60 b	28.22 ± 0.03 a	1427.47 ± 0.53 b	4.11 ± 0.03 a	6.17 ± 0.06 b
*Atriplex canescens*	0.16 ± 0.05 a	521.52 ± 0.09 b	1.38 ± 0.06 a	862.23 ± 0.04 b	2.09 ± 0.09 a	947.38 ± 0.97 b	2.35 ± 0.06 a	1454.92 ± 0.91 b	0.37 ± 0.05 a	1734.59 ± 0.07 b

Note: Two different lowercase letters in the same row of one metallic column indicate a significant difference (*p* < 0.01) between the treatments.

**Table 3:** Biological accumulation coefficient (BAC) of three desert plants.

	Cadmium		Nickel		Copper		Zinc		Lead	
	CK	T	CK	T	CK	T	CK	T	CK	T
*Suaeda glauca*	0.01	0.25	0.03	0.48	0.02	**1.05**	0.01	**1.03**	0	0.50
*Atriplex canescens*	0.03	**1.06**	0.05	**1.30**	0.08	**1.01**	0.04	**1.06**	0.02	**1.25**
*Artemisia desertorum*	0.26	0.33	0.44	0.51	0.33	**1.03**	0.48	**1.04**	0.22	0.33

Notes: Data with a BAC value greater than 1 are shown in bold.

## Data Availability

Not applicable.

## Supplementary Materials

The following supporting information can be downloaded at: https://www.mdpi.com/article/10.3390/ijerph192316035/s1, Table S1: The background value of heavy metals in the mining tailings.
