# Supplementary material for: Phytoremediation Potential and Physiological Mechanisms Underlying Metallic Extraction of Suaeda glauca, Artemisia desertorum, and Atriplex canescens
Source: Int J Environ Res Public Health. 2022 Nov 30;19(23):16035. doi: 10.3390/ijerph192316035 (PMC9736508; doi:10.3390/ijerph192316035)
Supplement: Supplementary file 1 [file ijerph-19-16035-s001.zip › ijerph-1963176-supplementary.pdf]

Supplement Table S1.

The background value of heavy metals in the mining tailings.

	Cd	Cu	Ni	Pb	Zn
			mg/kg soil		
Tailings	600.00	1530.00	156.00	4718.00	31736.00
